# The Time of the Hypertonic Saline Infusion Test for the Diagnosis of AVP Deficiency Can Be Shortened With LC-MS/MS

**DOI:** 10.1210/clinem/dgaf432

**Published:** 2025-07-30

**Authors:** Tomoko Handa, Daisuke Hagiwara, Ryutaro Maeda, Takashi Miyata, Tomoko Kobayashi, Mariko Sugiyama, Takeshi Onoue, Shintaro Iwama, Hidetaka Suga, Ryoichi Banno, Yachiyo Kuwatsuka, Hiroshi Arima

**Affiliations:** Department of Endocrinology and Diabetes, Nagoya University Graduate School of Medicine, Nagoya 466-8550, Japan; Department of Clinical Research Education, Nagoya University Graduate School of Medicine, Nagoya 466-8550, Japan; Department of Endocrinology and Diabetes, Nagoya University Graduate School of Medicine, Nagoya 466-8550, Japan; Department of Endocrinology and Diabetes, Nagoya University Graduate School of Medicine, Nagoya 466-8550, Japan; Department of Endocrinology and Diabetes, Nagoya University Graduate School of Medicine, Nagoya 466-8550, Japan; Department of Endocrinology and Diabetes, Nagoya University Graduate School of Medicine, Nagoya 466-8550, Japan; Department of Endocrinology and Diabetes, Nagoya University Graduate School of Medicine, Nagoya 466-8550, Japan; Department of Endocrinology and Diabetes, Nagoya University Graduate School of Medicine, Nagoya 466-8550, Japan; Department of Endocrinology and Diabetes, Nagoya University Graduate School of Medicine, Nagoya 466-8550, Japan; Department of Endocrinology and Diabetes, Nagoya University Graduate School of Medicine, Nagoya 466-8550, Japan; Department of Endocrinology and Diabetes, Nagoya University Graduate School of Medicine, Nagoya 466-8550, Japan; Research Center of Health, Physical Fitness and Sports, Nagoya University, Nagoya 464-0805, Japan; Department of Advanced Medicine, Nagoya University Hospital, Nagoya 466-8550, Japan; Department of Endocrinology and Diabetes, Nagoya University Graduate School of Medicine, Nagoya 466-8550, Japan

**Keywords:** arginine vasopressin, arginine vasopressin deficiency, liquid chromatography–tandem mass spectrometry, radioimmunoassay, hypertonic saline infusion test, regression analysis

## Abstract

**Context:**

In clinical practice, plasma arginine vasopressin (AVP) concentrations have been measured with a radioimmunoassay (RIA). However, RIAs have limitations, such as long turnaround time, use of radioisotopes, and restricted antibody availability. Liquid chromatography-tandem mass spectrometry (LC-MS/MS) offers a promising alternative, eliminating the need for radioisotopes and antibodies while providing faster results.

**Objective:**

This study aimed to assess the usefulness of LC-MS/MS for measuring plasma AVP concentrations in diagnosing AVP deficiency (AVP-D).

**Methods:**

We included 16 patients with AVP-D and 28 controls. All participants underwent a hypertonic saline infusion test (HST), during which plasma AVP concentrations were measured using RIA and LC-MS/MS. Regression coefficients (gradients) for serum sodium vs plasma AVP concentrations were evaluated at 90 and 120 minutes, and receiver-operating characteristic analyses were performed based on these regression coefficients.

**Results:**

The area under the receiver-operating characteristic curve at 90 minutes was 0.97 (95% CI, 0.83-1.00) and 0.93 (95% CI, 0.80-0.98) for LC-MS/MS and RIA, respectively. A regression gradient cutoff with optimal values distinguished AVP-D from controls with a sensitivity of 100% in LC-MS/MS and RIA, whereas the specificity was 96% and 81% with LC-MS/MS and RIA, respectively. Sensitivity or specificity did not differ in 120 minutes between the 2 methods.

**Conclusion:**

LC-MS/MS demonstrated superior diagnostic accuracy for AVP-D at 90 minutes of HST, indicating that the HST time can be shortened from 120 to 90 minutes by measuring AVP with LC-MS/MS.

**Clinical trial registration:**

The study was registered with the University Hospital Medical Information Network (UMIN) registry (UMIN000043023).

The magnocellular neurons of the supraoptic and paraventricular nuclei of the hypothalamus synthesize arginine vasopressin (AVP), which is delivered through axonal transport to the posterior pituitary and then released into the systemic circulation to promote water reabsorption in the renal collecting ducts. AVP production and secretion are tightly regulated by plasma osmolality, maintaining water balance homeostasis, whereas AVP system impairment results in AVP deficiency (AVP-D), characterized by polydipsia and polyuria.

Copeptin has been used as a surrogate biomarker of AVP in Europe, where sensitive AVP measurement systems are not widely available (1). The half-life of copeptin, derived from the AVP precursor preprovasopressin, is approximately twice as long as that of AVP (26 vs 12 minutes) and is more stable than AVP, making it easier to measure (2). Thus, the hypertonic saline infusion test (HST) with copeptin measurements has been recommended for AVP-D diagnosis (3-5). The difference in half-life are not a problem when serum sodium concentrations are stable, and previous studies demonstrated >95% accuracy for AVP-D diagnosis using copeptin measurements (3). However, this difference in half-life could still theoretically lead to a dissociation between copeptin and AVP concentrations under hyperosmolar or hypoosmolar conditions, when plasma AVP concentrations change dynamically. In Japan, high-sensitivity AVP measurements using a radioimmunoassay (RIA) and HST have been used to diagnose AVP-D. During the HST, 5% sodium chloride is infused intravenously at a rate of 0.05 mL/kg/min for 2 hours, and serum sodium and plasma AVP concentrations are measured before and every 30 minutes after the injection (6, 7). Plasma AVP increases in response to increasing serum sodium concentrations in the control, whereas a blunted or absent AVP response indicates AVP-D (8). In our previous study, RIA-based plasma AVP measurement during HST achieved 100% sensitivity and 77% specificity for AVP-D diagnosis when the regression gradient of plasma AVP against serum sodium concentration is <0.1 (9). However, the RIA for AVP is labor-intensive and time-consuming, and RIAs are limited by antibody availability.

Generally, liquid chromatography-tandem mass spectrometry (LC-MS/MS) offers advantages in specificity, selectivity, quantification accuracy, and safety compared with RIA (10-12). Moreover, it directly measures peptide masses and fragments, eliminating cross-reactivity issues inherent to antibody-based assays. Additionally, isotope-labeled internal standards in LC-MS/MS enhance quantification accuracy and reproducibility, whereas RIA may be affected by batch-to-batch variability in antibody production. LC-MS/MS does not require radioactive materials, unlike RIA, simplifying handling and disposal. Recently, plasma AVP concentrations have been measured using LC-MS/MS (13-15), with a detection limit of 0.2 pg/mL compared with RIA's 0.4 pg/mL (13). Thus, AVP can be reliably measured using LC-MS/MS; however, no studies have directly compared AVP concentrations using RIA and LC-MS/MS in clinically collected samples.

In this study, we performed the HST in patients with AVP-D and controls. RIA and LC-MS/MS were used to measure AVP concentrations to assess the usefulness of LC-MS/MS for AVP-D diagnosis.

## Material and Methods

### Patients

We enrolled 28 patients without AVP-D (control group; 13 males, 15 females) and 16 patients with AVP-D (AVP-D group; 6 males, 10 females). The mean age was 54.0 ± 2.7 and 54.2 ± 4.8 years in the control and AVP-D groups, respectively. The patients were classified as not having AVP-D if they met all the following criteria: (1) urine volume <3000 mL/day, (2) urine osmolality >300 mOsm/kg under euhydrated conditions or >600 mOsm/kg after a water deprivation test, and (3) presence of a hyperintense signal in the posterior pituitary on T1-weighted pituitary magnetic resonance imaging (MRI). AVP-D was diagnosed based on comprehensive clinical data, including urine volume, urine osmolality, absence of a hyperintense signal on T1-weighted pituitary MRI, response to 1-deamino-8-D-arginine vasopressin (DDAVP), and established diagnostic cutoff values for serum sodium and plasma AVP concentrations measured with the RIA during the HST (9). All participants were examined at Nagoya University Hospital between April 2021 and March 2024. The institutional review board of Nagoya University approved the study, and all participants provided written informed consent. The study was registered with the University Hospital Medical Information Network registry (UMIN000043023).

### HST

Before the HST, which commenced at 0900 hours, the participants remained in a supine position for 30 minutes. A 5% saline solution was administered at a rate of 0.05 mL/kg/min for 120 minutes. Blood samples were collected before and at 30, 60, 90, and 120 minutes after infusion. Samples were immediately placed on ice after collection and stored at 4 °C. Centrifugation was performed at 4 °C within 1 hour after completion of the HST to separate the serum and plasma, which were stored at −80 °C until analysis.

### Plasma AVP Measurement

RIA kit (Yamasa Cat# 80114, RRID:AB_2801274; YAMASA CORPORATION, Choshi, Japan) and LC-MS/MS with the JeoQuant Kit for AVP (JEOL Ltd., Tokyo, Japan) were used to measure plasma AVP concentrations during the HST. In the RIA method, AVP was extracted from the plasma using the cold ethanol method, with a 0.4 pg/mL detection limit (16). The LC-MS/MS assay used isotope dilution mass spectrometry with 300 µL plasma, achieving a lower limit of quantification of 0.2 pg/mL (13).

### Statistical Analysis

A simple regression line was calculated for each subject to assess the correlation between serum sodium and plasma AVP concentrations during the HST. The regression coefficients (gradients) were compared between the control and AVP-D groups. To assess the diagnostic accuracy, we performed receiver-operator characteristic (ROC) curve analyses and area under the curve calculations, with 95% CI using the DeLong method. Statistical analyses were conducted using GraphPad Prism 9 (GraphPad Software, Boston, MA, USA), Stata 18.0 (StataCorp LLC, College Station, TX, USA), and JMP Pro 17.2.0 (SAS Institute, Cary, NC, USA). All continuous variables are expressed as mean ± SE.

## Results

### Patients

In the control group, 22 of 28 patients (78.6%) had hypothalamic pituitary disorders, including nonfunctioning pituitary tumors (n = 6), acromegaly (n = 7), Cushing disease (n = 2), TSH-producing pituitary tumors (n = 2), empty sella (n = 1), adrenocorticotropic hormone deficiency (n = 3), and hypogonadism (n = 1) (Table 1). All cases had hyperintense signals on T1-weighted pituitary MRI.

**Table 1.** Characteristics of patients in the control group

Case no.	Gender	Age (y)	Diagnosis	Daily urine volume		Urine osmolality (mOsm/kg)	Timing of evaluation	Tumor size (mm)
				(mL)	(mL/kg)			
C1	M	68	Nonfunctioning pituitary tumor (apoplexy)	1700	25.6	849	—	21
C2	M	57	Acromegaly	1500	13.9	676	Before medical therapy	N/A
C3	M	60	N/A	1100	10.4	1151	—	N/A
C4	M	54	N/A	1150	14.9	486	—	N/A
C5	F	58	Nonfunctioning pituitary tumor	2800	38.5	372	—	18
C6	F	61	Cushing disease	1000	14.6	n.d.	Before TSS	12
C7	F	46	Cushing disease	1700	23.0	338	Before TSS	N/A
C8	M	59	Acromegaly	2400	35.9	577	Before TSS	9
C9	F	50	Acromegaly	ND	ND	594	Before TSS	10
C10	F	27	N/A	1500	34.9	ND	—	N/A
C11	F	51	Acromegaly	900	13.2	ND	Before TSS	23
C12	M	49	Empty sella	2800	32.0	186	—	N/A
C13	F	61	Acromegaly	840	18.3	807	Before TSS	41
C14	F	53	Acromegaly	2000	23.8	558	Before TSS	25
C15	F	78	N/A	1490	43.8	606	—	N/A
C16	F	79	ACTH deficiency	1000	21.1	453	—	N/A
C17	M	53	Nonfunctioning pituitary tumor	1170	18.5	825	Before TSS	29
C18	M	28	Hypogonadism	960	31.0	805	—	N/A
C19	M	33	TSH-producing pituitary tumor	700	13.6	817	Before TSS	42
C20	M	68	ACTH deficiency	2130	34.7	387	—	N/A
C21	F	55	Nonfunctioning pituitary tumor (recurrence)	2150	32.5	433	After TSS	25
C22	M	57	ACTH deficiency (irAE)	1120	17.1	851	—	N/A
C23	F	52	Nonfunctioning pituitary tumor	1960	31.9	592	Before TSS	25
C24	M	70	Nonfunctioning pituitary tumor	900	10.4	581	Before TSS	30
C25	F	18	N/A	800	14.6	ND	—	N/A
C26	F	48	Psychogenic polydipsia	4100	62.7	91	—	—
C27	F	64	Acromegaly	1260	23.4	525	Before TSS	15
C28	M	56	TSH-producing pituitary tumor (postoperative)	1860	30.2	534	After TSS	N/A

Abbreviations: F, female; irAE, immune-related adverse events; M, male; N/A, not applicable; ND, no data available; TSS, transsphenoidal surgery.

Three patients in the AVP-D group were previously diagnosed with AVP-D (Table 2). The remaining 13 patients were newly diagnosed during the study (yr = 0). The mean daily urine volume in the AVP-D group before DDAVP administration was 4960 ± 502 mL (85.4 ± 11.3 mL/kg) (n = 12), with a urine osmolality of 160.6 ± 26.0 mOsm/kg (n = 12). In the AVP-D group, 7 patients showed mild to moderate polyuria (<5000 mL/day) suggesting partial AVP-D, and 5 showed prominent polyuria (>5000 mL/day). All patients with AVP-D had diminished or no hyperintense signals on T1-weighted pituitary MRI. Seven patients had concurrent endocrine disorders, including secondary adrenal insufficiency and hypothyroidism, and they were started with hydrocortisone and thyroid hormone replacement before HST.

**Table 2.** Characteristics of patients in the AVP-D group

Case no.	Gender	Age (y)	Time after diagnosis (y)	Daily urine volume		Urine osmolality (mOsm/kg)	Etiology	DDAVP (μg)	Coexisting diseases
				(mL)	(mL/kg)				
AVP-D1	M	47	0	ND	ND	75	Nonfunctioning pituitary tumor (postoperative)	ODT (120)	—
AVP-D2	F	72	0	5500	100.2	96	Rathke's cleft cyst	ODT (60)	—
AVP-D3	M	73	0	4680	69.2	ND	Nonfunctioning pituitary tumor	ODT (60)	Panhypopituitarism
AVP-D4	M	40	0	ND	ND	ND	Nonfunctioning pituitary tumor (postoperative)	ODT (60)	Acromegaly, hypopituitarism (ACTH, TSH, LH, FSH, PRL)
AVP-D5	F	62	5	4500	72.5	19	Nonfunctioning pituitary tumor (postoperative)	ODT (60)	Cushing disease, hypopituitarism (TSH, LH, FSH, GH, PRL)
AVP-D6	M	54	0	4300	62.0	277	Rathke's cleft cyst	ODT (120)	Hypopituitarism (GH)
AVP-D7	F	18	5	7800	188.4	76	Germ cell tumor (after chemoradiation therapy)	ODT (360)	Panhypopituitarism
AVP-D8	F	81	0	3500	87.6	204	Idiopathic	ODT (60)	Pituitary microadenoma
AVP-D9	F	66	0	3350	74.9	203	Idiopathic	ODT (60)	—
AVP-D10	F	30	0	5270	66.0	106	Rathke's cleft cyst, Modest enlargement of the pituitary gland	ODT (120)	Hypopituitarism (GH)
AVP-D11	M	76	0	2770	65.8	246	Pituitary metastasis of salivary gland carcinoma	ODT (60)	Hypopituitarism (ACTH, TSH, LH, FSH, GH)
AVP-D12	M	76	0	ND	ND	ND	Nonfunctioning pituitary tumor (postoperative)	—	Panhypopituitarism
AVP-D13	F	57	0	6550	104.6	312	Rathke's cleft cyst	ODT (120)	Hypopituitarism (LH)
AVP-D14	F	33	0	8000	106.7	148	Rathke's cleft cyst (postoperative)	ODT (120)	Hypopituitarism (LH, FSH, GH)
AVP-D15	F	47	17	ND	ND	ND	Craniopharyngioma (postoperative)	ODT (120)	Hypopituitarism (ACTH, TSH, LH, FSH, GH)
AVP-D16	F	35	0	3300	26.9	165	Idiopathic	ODT (60)	Pituitary microadenoma

Abbreviations: AVP-D, arginine vasopressin deficiency; F, female; M, male; ND, no data available; ODT, orally disintegrating tablet.

### Plasma AVP Concentrations During HST Measured by RIA and LC-MS/MS

**Figure 1.**
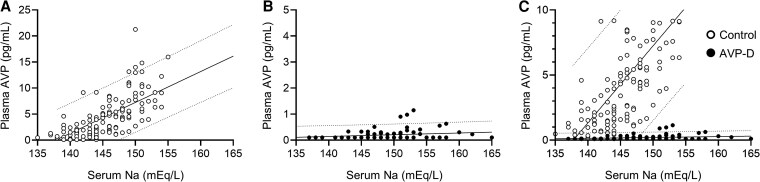
Serum sodium and plasma AVP concentrations during HST, with AVP measured using LC-MS/MS in the control (A, open circles, n = 28) and AVP-D groups (B, closed circles, n = 16) along with a comparison of both groups (C). The dashed lines indicate the 95% prediction intervals for the plasma AVP concentrations in each group.

**Figure 2.**
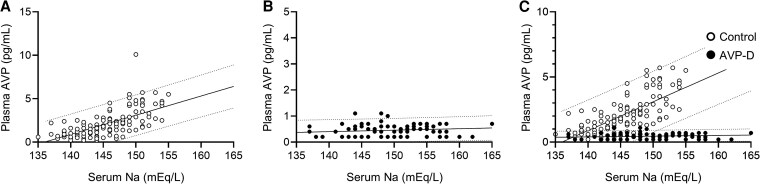
Serum sodium and plasma AVP concentrations during HST, with AVP measured using RIA. (A) Control group (open circles, n = 28). (B) AVP-D group (closed circles, n = 16). (C) Comparison of both groups. Dashed lines indicate the 95% prediction intervals for the plasma AVP concentrations in each group.

**Figure 3.**
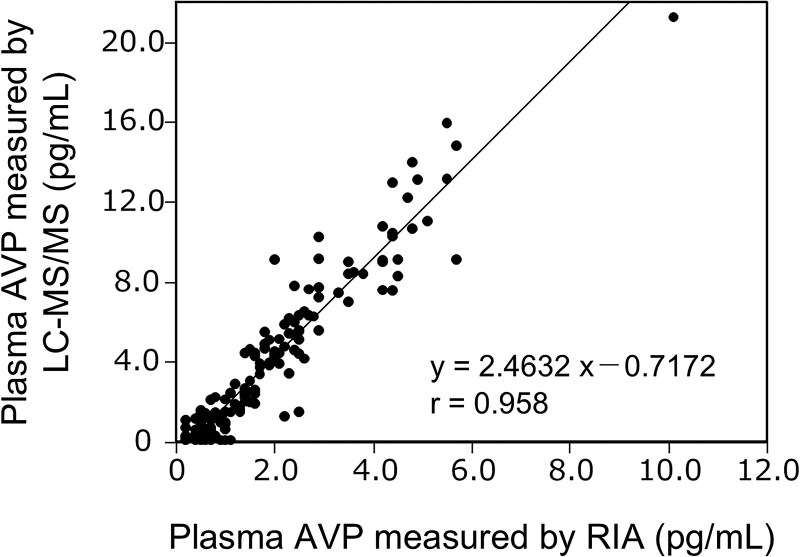
Correlation between AVP concentrations measured using RIA and LC-MS/MS during HST.

**Figure 4.**
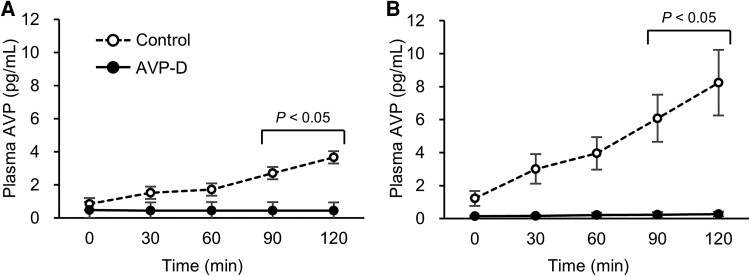
Changes in plasma AVP concentrations during HST in the control (open circles, dashed line) and AVP-D groups (closed circles, solid line), with AVP measured using RIA (A) and LC-MS/MS (B). The results are expressed as mean ± SE. *P* < .05 compared with the control group at each corresponding time point.

We presented serum sodium and plasma AVP concentrations measured by LC-MS/MS (Fig. 1; Table 3) and RIA (Fig. 2; Table 4) during HST. Plasma AVP concentrations measured by LC-MS/MS showed a strong correlation with those measured by RIA (*r* = 0.958, Fig. 3). Notably, LC-MS/MS yielded higher AVP values than the RIA, particularly at higher AVP concentrations (Figs. 3 and 4). Significant differences were observed in the plasma AVP concentrations at 90 and 120 minutes for each measurement method between the AVP-D and control groups during HST (Fig. 4).

**Table 3.** Serum sodium and plasma AVP concentrations measured by LC-MS/MS during HST in the control and AVP-D groups

	Control	AVP-D
Na (mEq/L) (0 minutes)	140 ± 3.0	144 ± 5.1
Na (mEq/L) (30 minutes)	144 ± 3.6	147 ± 5.1
Na (mEq/L) (60 minutes)	146 ± 3.5	150 ± 6.4
Na (mEq/L) (90 minutes)	148 ± 3.7	152 ± 5.9
Na (mEq/L) (120 minutes)	149 ± 3.4	153 ± 6.5
AVP (pg/mL) (0 minutes)	1.2 ± 0.2	0.1 ± 0.02
AVP (pg/mL) (30 minutes)	3.0 ± 0.4	0.2 ± 0.03
AVP (pg/mL) (60 minutes)	4.0 ± 0.5	0.2 ± 0.1
AVP (pg/mL) (90 minutes)	6.1 ± 0.7	0.2 ± 0.1
AVP (pg/mL) (120 minutes)	8.2 ± 1.0	0.3 ± 0.1

Results are expressed as mean ± SE.

Abbreviations: AVP, arginine vasopressin; AVP-D, arginine vasopressin deficiency; HST, hypertonic saline infusion test; LC-MS/MS, liquid chromatography-tandem mass spectrometry.

**Table 4.** Serum sodium and plasma AVP concentrations measured by RIA during HST in the control and AVP-D groups

	Control	AVP-D
Na (mEq/L) (0 minutes)	140 ± 3.0	144 ± 5.1
Na (mEq/L) (30 minutes)	144 ± 3.6	147 ± 5.1
Na (mEq/L) (60 minutes)	146 ± 3.5	150 ± 6.4
Na (mEq/L) (90 minutes)	148 ± 3.7	152 ± 5.9
Na (mEq/L) (120 minutes)	149 ± 3.4	153 ± 6.5
AVP (pg/mL) (0 minutes)	0.8 ± 0.1	0.5 ± 0.1
AVP (pg/mL) (30 minutes)	1.5 ± 0.1	0.4 ± 0.1
AVP (pg/mL) (60 minutes)	1.7 ± 0.2	0.5 ± 0.1
AVP (pg/mL) (90 minutes)	2.7 ± 0.3	0.5 ± 0.1
AVP (pg/mL) (120 minutes)	3.6 ± 0.4	0.4 ± 0.05

Results are expressed as mean ± SE.

Abbreviations: AVP, arginine vasopressin; AVP-D, arginine vasopressin deficiency; HST, hypertonic saline infusion test; RIA, radioimmunoassay.

### Comparison of Regression Line Gradients During HST Using RIA and LC-MS/MS

**Figure 5.**
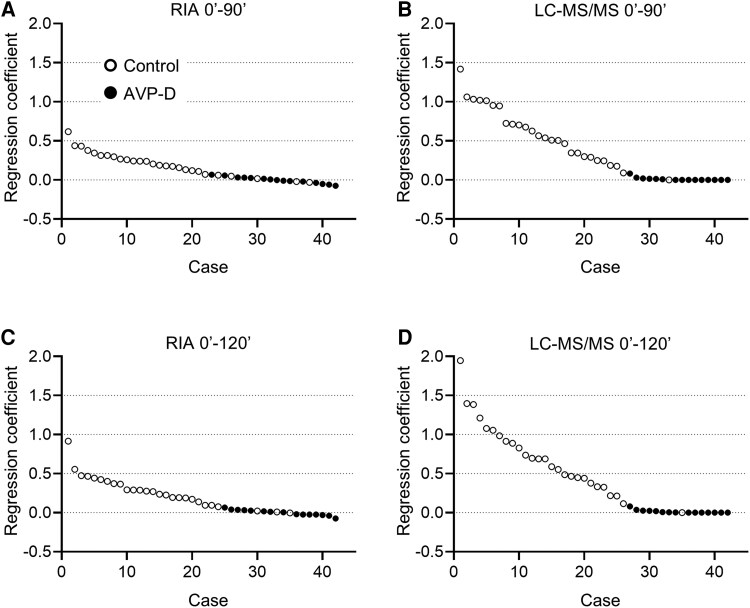
Regression coefficients (gradients) for serum sodium vs plasma AVP concentrations, measured using RIA and LC-MS/MS at 90 minutes (A, B) and 120 minutes (C, D) during HST in the control (open circles) and AVP-D groups (closed circles). The regression coefficients are arranged in descending order from left to right.

**Figure 6.**
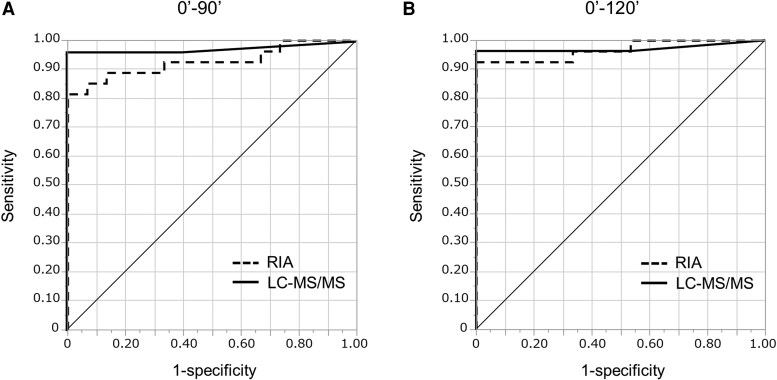
ROC curves of the regression coefficients (gradients) for AVP-D diagnosis using RIA (dashed line) and LC-MS/MS (solid line) based on HST data for 90 minutes (A) and 120 minutes (B).

Regression coefficients (gradients) for serum sodium vs plasma AVP concentrations measured using the RIA and LC-MS/MS were calculated at 90 (4 time points, Fig. 5A and B) and 120 minutes (5 time points, Fig. 5C and D) during HST. ROC analysis was performed to assess the diagnostic accuracy of AVP-D based on these regression coefficients (Fig. 6). The area under the curve of the ROC curve for HST at 90 and 120 minutes was 0.93 (95% CI, 0.80-0.98) and 0.97 (95% CI, 0.87-0.99) for the RIA method and 0.97 (95% CI, 0.83-1.00) and 0.97 (95% CI, 0.82-1.00) for the LC-MS/MS method, respectively (Table 5). A regression gradient cutoff value of 0.0673 at 90 minutes and 0.0656 at 120 minutes distinguished AVP-D from controls with a sensitivity of 100% and 100% and a specificity of 81% and 93% at 90 and 120 minutes using RIA, respectively (Table 5). Using a cutoff value of 0.0820 at 90 minutes and 0.0806 at 120 minutes, LC-MS/MS achieved 100% sensitivity and 96% specificity at 90 and 120 minutes (Table 5).

**Table 5.** The AUC of ROC curve of the regression coefficients (gradients) for AVP-D diagnosis using RIA and LC-MS/MS, based on HST data for 90 and 120 minutes

	AUC of ROC	95% CI	Sensitivity	Specificity	Optimal cutoff
RIA method (up to 90 minutes)	0.9284	0.7991-0.9769	1.0000	0.8148	0.0673
LC-MS/MS method (up to 90 minutes)	0.9741	0.8328-0.9965	1.0000	0.9630	0.0820
RIA method (up to 120 minutes)	0.9679	0.8653-0.9930	1.0000	0.9259	0.0656
LC-MS/MS method (up to 120 minutes)	0.9716	0.8187-0.9962	1.0000	0.9630	0.0806

Abbreviations: AUC, area under the curve; AVP-D, arginine vasopressin deficiency; HST, hypertonic saline infusion test; LC-MS/MS, liquid chromatography-tandem mass spectrometry; ROC, receiver operating characteristic.

### Adverse Events During and After the HST

Table 6 lists the adverse events during HST. A total of 25 adverse events occurred within 90 minutes of HST initiation, whereas 4 additional events (thirst, nausea, headache, and dizziness) were observed between 90 and 120 minutes. All events were tolerable.

**Table 6.** Adverse events in HST

	Control group (n = 28)	AVP-D group (n = 16)
Thirst	13 (46.4%)	6 (37.5%)
Nausea	2 (7.1%)	2 (12.5%)
Vomiting	0 (0%)	1 (6.3%)
Headache	2 (7.1%)	1 (6.3%)
Dizziness	1 (3.6%)	2 (12.5%)
Other	8 (28.6%)	4 (25.0%)
Fatigue	0 (0%)	1 (6.3%)
Vascular pain	2 (7.1%)	1 (6.3%)
Injection site pain	2 (7.1%)	0 (0%)
Upper extremity pain	2 (7.1%)	1 (6.3%)
Arm heaviness	1 (3.6%)	0 (0%)
Hand numbness	1 (3.6%)	1 (6.3%)

Abbreviation: HST, hypertonic saline infusion test.

## Discussion

We evaluated the plasma AVP concentrations in HST, a widely recognized method for assessing AVP secretion, using RIA and LC-MS/MS. Regression coefficients between serum sodium and plasma AVP concentrations were calculated at 90 and 120 minutes, and ROC analysis was used to assess the diagnostic accuracy for AVP-D. Our data showed that both measurements could be used to diagnose AVP-D. Notably, sensitivity or specificity did not differ between 90 and 120 minutes in LC-MS/MS. However, the specificity was lower at 90 minutes than at 120 minutes in RIA, indicating that 90 minutes is enough for HST when LC-MS/MS was used for AVP measurements.

The detection limit of AVP is 0.2 and 0.4 pg/mL with LC-MS/MS (13) and RIA, respectively (16). The time needed for the measurement with LC-MS/MS is a few hours (13), whereas it is several days for RIA. A rabbit polyclonal antibody is used in the YAMASA AVP RIA kit, and the reported cross-reactivity with structurally related peptides such as 8-arginine vasotocin, 8-lysine vasopressin, oxytocin, and DDAVP is 0.09%, 0.001%, <0.001%, and <0.001%, respectively (16). However, it is unclear if there is any cross-reactivity with AVP metabolites such as desglycinamide 9-AVP in the RIA. In contrast, AVP measurement using LC-MS/MS can distinguish compounds with different molecular masses from AVP (13), eliminating interference from structurally similar peptides such as DDAVP, 8-arginine vasotocin, 8-lysine vasopressin, oxytocin, and AVP metabolites. This analytical specificity represents a notable advantage of the LC-MS/MS method over RIA for measuring AVP.

A comparative analysis of AVP concentrations measured by RIA and LC-MS/MS demonstrated a strong correlation between both methods (Fig. 3). However, LC-MS/MS yielded higher AVP values than RIA at higher AVP concentrations (Fig. 3). The exact mechanisms underlying the discrepancy between LC-MS/MS and RIA remain unclear. Nonetheless, the AVP concentrations measured by LC-MS/MS were also approximately one-third of the undiluted values when the samples were diluted to one-third (Table 7), indicating the reliability of AVP measurement at a high concentration with LC-MS/MS. Our findings that LC-MS/MS can distinguish AVP-D from the control at 90 minutes in HST may be, at least in part, due to higher values of plasma AVP concentrations in LC-MS/MS than in RIA. Reducing HST time could lower the peak of sodium concentrations, thereby minimizing hypernatremia-related adverse events, such as thirst, which commonly occur during HST (3, 17). We observed four additional adverse events between 90 and 120 minutes. Thus, AVP measurements with LC-MS/MS would reduce the burden of patients with AVP-D during HST.

**Table 7.** AVP concentrations using LC-MS/MS when the sample was diluted 3-fold (n = 4)

	Undiluted (pg/mL)	3-fold diluted (pg/mL)
No. 1	10.12	3.05
No. 2	6.25	2.36
No. 3	10.86	4.26
No. 4	10.25	3.73

Abbreviations: AVP, arginine vasopressin; LC-MS/MS, liquid chromatography-tandem mass spectrometry.

The water deprivation test has traditionally been used for the differential diagnosis of polyuria-polydipsia syndromes (18). However, the water deprivation test requires a longer duration than the HST, and patients with AVP-D have been reported to prefer the HST (3). Moreover, copeptin measurements did not improve the diagnostic accuracy of the water deprivation test for AVP-D (3).

In previous studies (2, 3, 19), 3% saline was used for the HST, with an initial bolus of 250 mL followed by continuous infusion at 0.15 mL/kg/min; blood samples were collected every 30 minutes until the serum sodium concentrations exceeded 150 mEq/L. In contrast, the 2023 Japanese guidelines for the diagnosis and treatment of hypothalamic pituitary dysfunction recommends the use of 5% saline at 0.05 mL/kg/min for 2 hours without an initial bolus (8). This protocol gradually increases in serum sodium concentrations to approximately 150 mEq/L. Here, using 5% saline, the serum sodium concentration in the AVP-D group reached 152 ± 5.9 and 153 ± 6.5 mEq/L at 90 and 120 minutes, respectively (Tables 3 and 4). These values are comparable to those reported in studies that used 3% saline, in which serum sodium concentrations in patients with complete AVP-D reached approximately 153 mEq/L at approximately 80 minutes and those in patients with partial AVP-D reached approximately 152 mEq/L at approximately 100 minutes (3). Adverse events, such as nausea and vomiting, were observed with both protocols. Local vascular pain at the infusion site, potentially because of the high osmolarity of 5% saline, was reported in 1 patient in this study. It is of note that neither 3% nor 5% HST is suitable for pediatric subjects or for patients with impaired thirst sensation where serum sodium concentrations could be high even under basal conditions.

LC-MS/MS is a quantitative analytical technique highly sensitive and selective; it has been widely used to measure compounds, such as vitamin D, steroid hormones, immunosuppressants, and peptides (12, 20-23). However, the LC-MS/MS system is expensive and requires highly skilled personnel for its setup, operation, and maintenance. Thus, infrastructure and human resources limit the broad implementation of LC-MS/MS in clinical laboratories, particularly in developing countries. Moreover, the AVP in the serum is susceptible to enzymatic degradation, requiring careful sample handling before extraction, even if the measurement time is shorter in LC-MS/MS than in RIA. In contrast, the analyzers of copeptin are not available in some countries and the measurement is not covered by the Japanese national health insurance system. Thus, the choice of assays, AVP or copeptin, depends on their availability in each country.

This study has several limitations. First, although differentiating partial AVP-D from primary polydipsia is most challenging in clinical practice, only 1 patient with primary polydipsia was included in the control group. In addition, none of the participants had a water deprivation test in this study, whereas 7 patients showed mild to moderate polyuria (<5000 mL/day), so we are uncertain about how many patients with partial AVP-D were included in the AVP-D group. Second, this is the first pilot study involving a small sample size; larger studies with primary polyuria and partial AVP-D patients are warranted to confirm the findings. Third, we have not compared AVP measured by LC-MS/MS with copeptin, which is an important future direction.

In conclusion, AVP measurement using LC-MS/MS demonstrated high diagnostic accuracy for AVP-D at 90 minutes in HST, indicating that the HST time could be shortened by measuring the plasma AVP concentrations with LC-MS/MS.

## Data Availability

Some or all datasets generated during and/or analyzed during the current study are not publicly available but can be provided by the corresponding author upon reasonable request.

## Abbreviations

AVP: arginine vasopressin
AVP-D: arginine vasopressin deficiency
DDAVP: 1-deamino-8-D-arginine vasopressin
HST: hypertonic saline infusion test
LC-MS/MS: liquid chromatography-tandem mass spectrometry
RIA: radioimmunoassay
ROC: receiver-operator characteristic
